# Prostate immobilization using a rectal balloon

**DOI:** 10.1120/jacmp.v3i1.2590

**Published:** 2002-01-01

**Authors:** John. E. McGary, Bin S. Teh, E. Brian Butler, Walter Grant

**Affiliations:** ^1^ Department of Radiology Baylor College of Medicine Houston Texas 77030

**Keywords:** immobilization, prostate radiotherapy, organ motion

## Abstract

We use a rectal balloon for prostate immobilization during intensity modulated radiotherapy (IMRT) prostate treatment. To improve the accuracy of our prostate planning target volume, we have measured prostate displacements using computed tomography (CT)‐CT fusion on patients that previously received gold seed implants. The study consists of ten patients that were scanned twice per week during the course of IMRT treatment. In addition to biweekly scans, breathing studies were performed on each patient to estimate organ motion during treatment. The prostate displacement in the anterior‐posterior and the lateral direction is minimal, on the order of measurement uncertainty (~1mm). The standard deviation of the superior‐inferior (SI) displacements is 1.78 mm. The breathing studies show that no organ displacement was detected during normal breathing conditions with a rectal balloon.

PACS number(s): 87.53.–j, 87.90.+y

## INTRODUCTION

The goal of intensity modulated radiotherapy (IMRT) is to minimize the dose to normal tissue surrounding the clinical target volume (CTV). The planning target volume (PTV) is defined to include the CTV and associated treatment uncertainties, which include, but are not limited to imaging, patient setup and organ motion. For conditions where planning margins are not sufficient, the tumor will be underdosed. In contrast, margins that are too large may lead to greater complications. While it is impossible to eliminate these errors, the goal is to measure the planning margins for a specific clinical environment and reduce the uncertainties where possible.

Prostate motion, defined as a positional change of the prostate at the time of treatment relative to the planning position, has been evaluated for different conditions and methods.^1^ ^–^ ^11^ Radio‐opaque markers, gold seeds, computed tomography (CT)‐CT fusion, and CT chamfer matching are examples of methods used to determine the prostate position relative to fixed bony landmarks. Within these studies, the largest motion was observed to be in the anterior‐posterior (AP) direction and to a lesser extent in the superior‐inferior (SI) direction. The variation in AP data between reports is considerable. Values of A/P shifts range from (−0.9‐mm mean, 1.7‐mm standard deviation) to (−5.4‐mm mean, 6.2‐mm standard deviation). The range in S/I shifts were (−0.2‐mm mean, 3.2‐mm standard deviation) to (−5.9‐mm mean, 5.0‐mm standard deviation). The lateral displacements were shown to be minimal–the mean and standard deviations were less than 1.5 mm. In addition to measuring organ movement, correlations were made for bladder and rectal filling, rectal contrast, and random bladder volumes. A/P movement was strongly correlated with rectal filling, and then to a lesser extent with bladder filling. In the case of patients treated in the prone position, the bladder effect was correlated with both AP and SI movement.^1^ A current summary of setup errors and organ motion results is found in Antolak *et al.* ^11^

In this paper, we present prostate displacement measurements associated with a rectal balloon to identify immobilization uncertainties expected for our IMRT treatment setup. We use gold prostate seed implants as markers to estimate organ motion.

## METHODS AND MATERIALS

### A. Treatment setup review

The details of patient setup and the associated setup errors have been described previously^12^ and therefore we will present only the basic patient setup here. Patients are setup in the prone position and immobilized using a Vac‐Lok™ bag (MED‐TEC, Orange City, IO), which is fitted to a plywood box designed for registration and support. The carrier box is designed to maintain the shape of the Vac‐Lok bag for patient repositioning and prevent break down over extended periods of time. For the planning CT, the patient enters the box with a preformed Vac‐Lok bag. The bag is then partially deflated and reformed with the patient in the box. After the bag formation procedure is finished and the patient is placed in the treatment position, a rectal catheter (a barium enema tip typically used for radiological procedures) is inserted and inflated to 100 cm^3^. Approximately 5–10 minutes is allowed to pass for the patient to adapt to the catheter before performing the CT. The goal is to relax the patient as much as possible before scanning. After the CT is performed, the patient's legs are rolled laterally, and three continuous, vertical marks were drawn from the calves to the bag. The treatment position and leg marks are recorded and the patient is released.

Patients are treated with the NOMOS (Sewickley, PA) MIMiC (multileaf intensity modulating collimators) radiation delivery system. Patients climb into the prostate box, which is set on the treatment table, and are coached into position according to leg and/or bag marks and pictures from the original planning setup. The legs are rolled laterally to loosen skin contact and the final position is confirmed with setup pictures made during the original CT scan. The rectal catheter is then inserted and inflated. After registering the patient to the box, the box is aligned to the treatment room lasers using the treatment alignment box. The alignment box consists of fiducials for both lateral lasers and an overhead sagittal laser that maintains alignment within 2 mm for each direction. After alignment, the leg marks are checked again for movement.

### B. Rectal balloon

The rectal catheter is a catheter with an inflatable balloon (E‐Z‐EM, Westbury, NY) that is typically used for radiology studies. For immobilization purposes, the balloon is first tested for leaks by inflating the balloon with approximately 50 cm^3^ of air and waiting for a minute. The catheter is then enveloped with a condom sheath. Lubricating jelly (K‐Y brand, Johnson & Johnson Medical, Arlington, Texas) is applied to coat the condom sheath. After the catheter is lubricated, the ensheathed catheter is then inserted gently and carefully into the anorectum. The balloon is then inflated with 100 cm^3^ of air for the daily treatment. After completing the daily radiation treatment, the balloon is deflated and gently removed. As the patient is treated in the prone position and with a rectal balloon, a full bladder is very uncomfortable and is not set as a requirement. Rectal catheters are replaced every two weeks to avoid potential problems that may occur from slow leaks from damage to the balloon.

### C. Prostate displacement measurements

Patients undergoing IMRT treatment with previous gold seed implants were selected for these studies, since the gold seeds serve as markers to identify the prostate organ. Each prostate patient had 40 seeds implanted within the prostate two weeks before external beam IMRT with a standard seed dimension of 0.8‐mm diameter×2.5‐mm length. Ten patients were scanned twice a week during the course of treatment to determine the prostate displacements and better estimate the prostate immobilization uncertainties. Patients were scanned in the treatment position with the rectal balloon inflated. Ten minutes were allowed to pass before scanning to simulate the treatment environment in terms of patient comfort and stability within the box. Each CT scan set was fused with the planning CT set using the Marconi VoxelQ Fusion software package (Marconi Medical Systems Inc., Highland Heights, OH).

**Figure 1:**
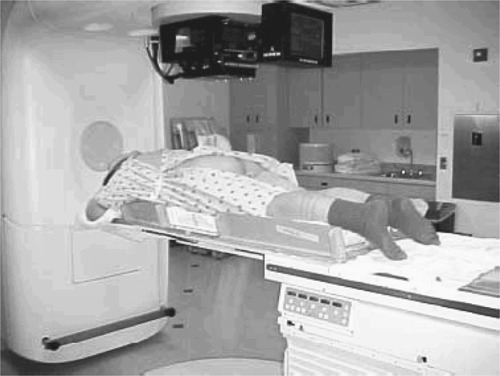
Treatment setup.

**Figure 2:**
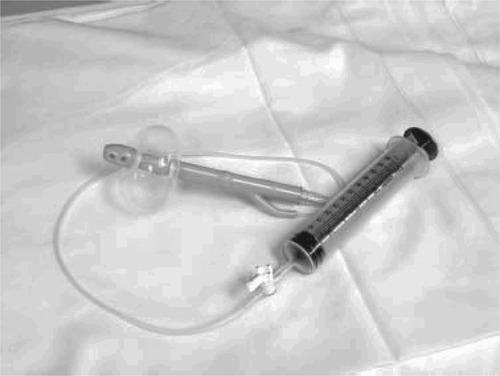
Rectal ballon.

CT data sets were registered according to bony landmarks. Three seed groups were chosen for comparison to avoid registration problems due to seed migration. At least two seeds in the inferior, mid, and superior aspects of the prostate were used for measurements and analysis. The scanning parameters used for these studies were 1 mm for slice index and thickness with a field of view that produced a 0.48‐mm pixel size. Anterior‐posterior and lateral displacements were measured within the axial slices with an associated uncertainty due to the pixel resolution of ~0.5 mm. For estimating the superior‐inferior displacements, the interslice spacing between seed centers was used where the associated errors were determined to be ~1mm.

In addition, breathing studies were performed after the displacement study CT scans were taken while the patient remained in the treatment position. CT images were taken at a single fixed couch position to examine the prostate motion in one slice. Over a period of ten minutes, axial scans were acquired at intervals varying from 15–40 seconds. The resulting CT axial images were compared to determine the AP and lateral motion as a function of breathing. To determine the SI breathing motion, the axial image was compared with images taken from the previous CT scan set.

## RESULTS AND DISCUSSION

The largest prostate displacements were observed in the superior‐inferior (SI) direction. Typically, the SI prostate displacement for a patient was ~1mm with several displacement measurements that ranged between 3 and 4 mm. In contrast, the anterior‐posterior and lateral displacements were consistently less than 1 mm. Table I summarizes the displacement measurements for the ten patients in the study.

**Table I:** Summary prostate displacement.

	Mean (mm)	Standard deviation (mm)
Anterior‐posterior	0.42	0.35
Lateral	0.83	0.38
Superior‐inferior	0.92	1.78

To eliminate seeds from the analysis that might have migrated over the course of treatment, the seeds used for displacement measurements were taken from different prostate regions. Groups of two were selected from the inferior, midsection, and superior regions of the prostate. The displacements were then determined from the average of the seed displacement measurements. Seeds were to be eliminated from the analysis if a seed displacement exceeded the average displacement of all the seeds by more than 1 mm. However, no seed migration (>1mm) was observed in this study.

The superior‐inferior displacement measurements exhibited the most uncertainty. Measuring displacements for conditions where the prostate moved were difficult due to the uncertainty presented within the slice thickness and identifying the seed centers between images. The uncertainty in these measurements was estimated to be ~1mm.

For normal breathing, the prostate was not detected to move relative to the bony landmarks as a function of breathing. The gold seeds were used to measure the prostate displacement within the axial images, for a fixed couch position, where the anterior‐posterior and lateral displacement were measured to be less than 0.5 mm. The rectal balloon effectively immobilizes the prostate in these two directions. However, when the patients were instructed to breathe deeply, the patients moved within the box. It was difficult to assess the actual movement but the axial images were compared with the previous CT scan set to estimate the skeletal motion. It was estimated that patient movement under these conditions was 2‐3 mm, however, the prostate remained fixed relative to the pelvic bones.

Determining the SI prostate movement during breathing is difficult to analyze since the seeds are ~3mm in length and can possibly span three 1‐mm thick axial slices if the length of the seed is aligned normal to the scan axis. Slices that contain several seeds reduces the uncertainty presented when using one seed since the orientation varies among seeds and are not usually normal to the axial plane. Furthermore, the relative positions of the seeds along the body axis are different and exhibit different intensities. As an example, the intensity may be less near the seed tip than within the center of the seed. An increment in the couch of 1 mm may show one seed to become brighter while the other remains unchanged. This indicates motion but is difficult to determine the displacement accurately. From the displacement CT scan of the previous study, we estimated the maximum uncertainty for not detecting displacements by inspecting adjacent axial images. We found that the maximum distance between slices where the seeds appeared to be the same (intensity and position) was 2 mm. With respect to the normal breathing study, the intensity and location of the seeds did not change between axial slices but may have moved 2 mm without detection.

In comparison with breathing studies for unimmobilized prostate conditions, the rectal balloon is a major improvement. A previous investigation reported that the prostate moves substantially for patients setup in the prone position and less in the supine position.^13^ For prone setup, the prostate was observed to move 5.1 mm in the SI direction and 3.5 mm in the AP direction. In comparison, prostate motion was observed to be negligible, ~1mm for supine setups. Deep breathing was reported to significantly affect prostate motion. For prone setup, the SI range of motion extended to 6–10 mm, whereas the range was reduced to 2–7 mm in the supine position.

Traditional planning target volume margins for the prostate range from 5 to 15 mm without considering the ventilatory prostate movement which can be comparable in magnitude.^13^ Not only will breathing increase the PTV margin, but the associated motion artifacts on the planning CT studies will decrease the target definition.^14^ These effects are critical for intensity modulated radiotherapy. In contrast, the rectal balloon essentially eliminates many of these problems by immobilizing the prostate during CT planning and radiotherapy treatment. With respect to our treatment setup method, the balloon reduces the PTV by 5 mm in the anterior‐posterior direction, 1 mm in the lateral direction, and 2 mm in the superior‐inferior direction. The most significant problem for reducing the PTV resides in the superior‐inferior setup error which is ~10.5 mm and the prostate motion is almost negligible in comparison.

In comparison with previous motion studies, the rectal balloon offers the most improvement in the anterior‐posterior direction where unimobilized prostate planning target volume margins due to organ motion range from 4–12 mm. The improvement in the superior‐inferior direction is less where the rectal catheter reduces the margins by 2–6 mm. Lateral displacements are typically small and the rectal balloon retains that effect without a significant decrease.

Our initial experience with the patients’ response to the use of rectal balloon has been encouraging. Over 400 patients were treated with IMRT using the rectal balloon for prostate immobilization. About half the patients had no problem with the procedure, the other half stated that there was some degree of discomfort but the procedure was tolerable and would do it again. No patient claimed that rectal balloon caused too much discomfort that they would not do it again although two patients could not tolerate 100 cm^3^ of air and the volume of air in the balloon was reduced to 50 cm^3^. We have also observed that the treating therapists play an important role. The patients had reported different comfort levels among the treating therapists. When the therapists take extra care and do not perform the procedure too hastily, the tolerance of the patients increases.

## CONCLUSION

Using a rectal balloon immobilizer for prostate IMRT reduces prostate motion, both in terms of interfraction organ displacement and movement due to breathing. With respect to interfraction prostate displacement, the anterior‐posterior (AP) organ displacement is <1mm and the cranial‐caudal displacement range is less than 5 mm. In addition, ventilatory movement of the prostate is essentially eliminated with respect to skeletal motion. To date, patients have tolerated the balloon without much discomfort and the use of the rectal balloon has been shown to be clinically viable.
